# Case report: an infant with late-onset meningitis caused by Escherichia coli

**DOI:** 10.3205/dgkh000522

**Published:** 2024-12-16

**Authors:** Aria Asghari, Saeed Khoshnood, Zahra Mousavi, Hamid Heidari, Farzaneh Peik Falak, Farhad Dadgar, Hossein Ali Rahdar, Hossein Kazemian

**Affiliations:** 1 Department of Medical Genetics, School of Medicine, Iran University of Medical Sciences, Tehran, Iran; 2 Clinical Microbiology Research Center, Ilam University of Medical Sciences, Ilam, Iran; 3 Students Research Committee, Ilam University of Medical Sciences, Ilam, Iran; 4 Department of Microbiology, Faculty of Medicine, Shahid Sadoughi University of Medical Sciences, Yazd, Iran; 5 Department of Internal Medicine, Iranshahr University of Medical Sciences, Iranshahr, Iran; 6 Department of Rheumatology, Iranshahr University of Medical Sciences, Iranshahr, Iran; 7 Department of Microbiology, School of Medicine, Iranshahr University of Medical Sciences, Iranshahr, Iran

**Keywords:** meningitis, Escherichia coli, infant, cerebrospinal fluid, neonatal intensive care unit

## Abstract

**Background:**

Meningitis is highly prevalent in infant because their immune system is immature and they have less resistance to diseases. Among bacterial agents, *Escherichia coli* is recognized as one of the most important causes of meningitis in infants.

**Case presentation:**

Herein, we report a case of late-onset meningitis, caused by *E. coli* (Patient:17-day-old female infant). The patient’s body temperature was 39°C, and the initial diagnosis was sepsis. At the doctor’s request, the patient underwent the basic tests and was hospitalized in the Neonatal Intensive Care Unit (NICU). In this case, blood culture and CSF culture were negative and positive, respectively. Echogenic particles were observed inside the bladder, indicating possible cystitis. The results of the antibiotic susceptibility tests showed that the meningitis-causing strain of *E. coli* was susceptible only to amikacin.

**Conclusion:**

Conducting LP and CSF culture seems to be the most important strategy for diagnosing meningitis. It is also recommended to perform LP before taking antibiotics. For identifying the infection, some factors such as fever, CRP test results, CSF parameters (leukocyte count, glucose level, and CSF culture results) should be considered to prevent misdiagnosis.

## Zusammenfassung

**Einführung:**

Hirnhautentzündungen treten besonders häufig bei Kleinkindern auf, da ihr Immunsystem noch nicht ausgereift ist und sie weniger widerstandsfähig gegen Krankheiten sind. Unter den bakteriellen Erregern gilt *Escherichia coli* als eine der wichtigsten Ursachen für Meningitiden bei Säuglingen.

**Fallbeschreibung:**

Wir berichten über einen 17 Tage alten Säugling mit late-onset Meningitis, die durch *E. coli* verursacht wurde. Die Körpertemperatur betrug 39°C; die erste Diagnose lautete Sepsis. Auf Ersuchen des Arztes wurde die Patientin zu den grundlegenden Untersuchungen und zur stationären Aufnahme in die Neugeborenen-Intensivstation gebracht. Die Blut- und die Liquorkultur waren negativ bzw. positiv. In der Blase wurden echogene Partikel beobachtet, die auf eine Zystitis hinweisen können. Die Ergebnisse des Antibiogramms zeigten, dass der *E. coli*-Stamm nur gegen Amikacin empfindlich ist.

**Schlussfolgerung:**

Die Durchführung einer Liquorkultur scheinen die wichtigsten Strategien für die Diagnose einer Meningitis zu sein. Es wird auch empfohlen, vor der Einnahme von Antibiotika eine Liquorpunktion durchzuführen. Zur Identifizierung der Infektion sollten Faktoren wie Fieber, C-reaktives Protein, Liquor-Parameter (Gesamtleukozytenzahl, Glukosespiegel und Liquor-Kulturergebnisse) berücksichtigt werden, um Fehldiagnosen zu vermeiden.

## Introduction

Meningitis is a severe infection and inflammation of the meninges that covers the central nervous system. Bacteria, viruses, fungi and parasites are among the factors causing meningitis. Autoimmune diseases and cancer are also effective in the occurrence of this infection [1]. Meningitis is a common infection among infants, owing to their immature immune system and low resistance to diseases [2]. Infant meningitis is divided into two types: early-onset and late-onset, which occur first 72 hours of life and after first 72 hours of life, respectively [3]. Among bacterial agents, *Escherichia (E.) coli* has been known as a major cause of meningitis in infants, so that after group B streptococcus, it is the second cause of premature meningitis in infants. It is also the third cause of late-onset meningitis in infants after coagulase-negative *Staphylococcus* and *Staphylococcus* *aureus* [4]. Herein, we report a case (infant) of late-onset meningitis, caused by *E. coli*, with negative blood culture and positive cerebrospinal fluid (CSF) culture.

## Case report

The patient is a 17-day-old female infant, weighing 3,120 g, who came with the symptoms of fever and restlessness due to an unknown cause. The patient’s body temperature was 39°C, and the doctor’s initial diagnosis was sepsis. At the request of the doctor, the patient was taken to the basic tests and hospitalization at the Neonatal Intensive Care Unit (NICU). Based on the patient’s weight, 150 mg of ceftazidime, 45 mg of vancomycin, and 45 mg of intravenous (IV) Acetaminophen (Apotel^®^) were prescribed as IV and STAT (abbreviation of the Latin word “statim,” meaning “immediatel”), to start the initial treatment until the cause of the disease was determined. The results of initial (hematology, biochemistry, urine, and serology) tests on the first day of hospitalization are listed in Table 1 (Tab. 1). On the first day of hospitalization, the patient received 150 mg of cefepime IV and STAT administration. The results of 48-hour blood culture and urine culture of the patient were negative. On the second day of hospitalization, CSF analysis and culture were performed. On the fourth day of hospitalization, the result of culture and analysis of CSF showed growth of a Gram-negative bacterium, which after performing differential tests, the result was positive for *E. coli* (Table 1 (Tab. 1)). 

The results of the antibiogram showed that this strain was sensitive to only amikacin. Considering the report of these results on the fourth day of hospitalization, the patient was prescribed 45 mg of amikacin by iv route daily, and the results of the antibiogram for sensitivity was the same. After reporting these results on the fourth day of hospitalization, 45 mg of amikacin was again administered IV to the patient daily. On the eighth day of hospitalization, CSF analysis and culture were performed for the second time (Table 1 (Tab. 1)). The result was negative, and the bacteria did not grow. In addition, an ultrasound of the kidney and urinary tract was taken from the patient. The results of imaging test showed that the two kidneys were normal in size. Also, the kidneys were visualized with echo parenchyma, and cortical thickness and pyelocaliceal system were normal. There was no sign of stone or lesion in the kidney. The patient's bladder had a normal wall thickness. Echogenic particles were observed inside the bladder, which can indicate cystitis.

On the 10^th^ day of hospitalization, a brain ultrasound was taken, who showed that the extra-axial space and cerebral ventricles had normal dimensions and appearance, and there was no evidence of ventriculomegaly and hydrocephalus. Brain parenchyma, including white and gray matter, was normal according to echo. The posterior cavity, comprising the brain and cerebellum, had a normal appearance. No evidence of periventricular leukomalacia or hematoma was found. On the 12^th^ day of hospitalization, the patient went on temporary leave at the request and consent of the family and visited daily the hospital to receive medicine. On the 20^th^ day of hospitalization, the patient was discharged from the hospital with good general condition and tolerance of per os (PO) (ability to eat by mouth).

## Discussion

Bacterial meningitis is one of the most important reasons for meningitis in infants and can lead to severe disabilities in those who survive and result in severe disorders [1]. Bacterial meningitis can induce complications between 20% and 60% [5]. Research has shown that meningitis accounts for 3% of all deaths of infants under five years of age, and mortality from this infection among infants under five years of age is more than other age groups [1]. One of the predisposing factors for infants is the immaturity of their immune system, which does not reach maturity until two months [2]. The death rate from meningitis in one-month-old infants who were treated reaches 40% [2]. Up to 50% of survived infants suffer from neurological complications, such as seizures, cognitive defects, movement problems, and hearing and vision disorders [4], [6], [7], [8]. Moreover, the death rate from neonatal meningitis is 10% in developed countries and 40–58% in developing countries [6]. All these data show the importance of meningitis in infants and make it one of the most dangerous diseases of infants. 

*E. coli* is one of the most important causes of meningitis, as well as the second and the third cause of early-onset and late-onset meningitis in infants, respectively [4]. It is also a major cause of meningitis is in infants born with low weight (less than 1,500 g). Evidence has revealed that *E. coli* meningitis increases the risk of relapsing infection in infants [9]. In this study, we investigated a 17-day-old infant with late-onset neonatal meningitis. The patient was admitted with the symptoms of sepsis, and necessary tests were performed. The infant had symptoms of fever and restlessness and irritability, which are among the signs and symptoms of meningitis [2]. The infant’s body temperature at admission was 39°C. However, it is recommended that infants who are less than 28 days old, if they have a fever of 38 °C or higher, sepsis should be suspected in the newborns and the necessary tests should be considered. 

In the patient's basic tests, CRP was higher than the normal range. High CRP is one of the symptoms of serious bacterial infection in infants [2]. The patient's blood culture was examined to determine the cause of the disease, and the culture result was negative. Research has demonstrated that in 15–38% of cases, blood cultures may be negative in infants with meningitis [4]. For CSF analysis, we performed lumbar puncture (LP), as well. The result of culture was positive for *E. coli*, and the CSF analysis showed a very low number of leukocytes. According to research, analyzing CSF and leukocyte number is one of the most significant diagnostic factors of meningitis [10]. Some research has also reported that meningitis can occur with normal levels of leukocytes [4]. Another important factor in CSF is the level of glucose; the decrease of which indicates a bacterial infection [11]. 

At the beginning of our study, patient was suspected to have blood infection; as a result, a sample was obtained from the patient for blood culture. At the same time, the patient started taking antibiotics to prevent the disease from progressing. However, a number of researches believed in performing LP at the beginning and before the initiation of antibiotics consumption because the use of antibiotics causes false-negative results and makes diagnosis difficult [2]. After determining the cause of the disease and performing an antibiogram, we found that the second culture of the patient's CSF was negative due to the use of antibiotics. Polymerase chain reaction (PCR) is one of the most sensitive methods for the diagnosis of meningitis [2] and can identify pathogenic pathogens when antibiotics are used [4]. However, more studies are needed to use PCR for diagnosis, and CSF culture is still considered the best method to diagnose meningitis though [2]. 

The cause of late-onset meningitis in the patient is unknown. Some studies consider poor hygiene among health care workers and hospital staff as the cause of transmission. In cases where the gestational age was less than 32 weeks, the probability of the baby suffering from late-onset sepsis is higher [4].

## Conclusion

Conducting LP and CSF culture seems to be the most important strategy for diagnosing meningitis. It is also recommended to perform LP before taking antibiotics. For identifying the infection, some factors such as fever, CRP test results, CSF parameters (WBC numbers, glucose level, and CSF culture results) should be considered to prevent misdiagnosis. The PCR method can also be a suitable diagnostic method with further studies.

## Notes

### Consent for publication 

Written informed consent was obtained from the patient’s legal guardian for publication of this case report and any accompanying images. A copy of the written consent is available for review by the Editor-in-Chief of this journal.

### Authors’ ORCID

- Asghari Aria: 0000-0002-7914-5290
- Khoshnood Saeed: 0000-0002-5143-3178
- Mousavi Zahra: 0000-0002-3645-9724
- Heidari Hamid: 0000-0002-6869-2301
- Peik Falak Farzaneh: 0000-0001-8967-6805
- Dadgar Farhad: 0000-0001-5487-2339
- Ali Rahdar Hossein: 0000-0003-1583-9936
- Kazemian Hossein: 0000-0003-4590-396X

### Author contributions 

The authors Asghari A and Khoshnood S have contributed equally to this work.

### Competing interests

The authors declare that they have no competing interests.

## Figures and Tables

**Table 1:**
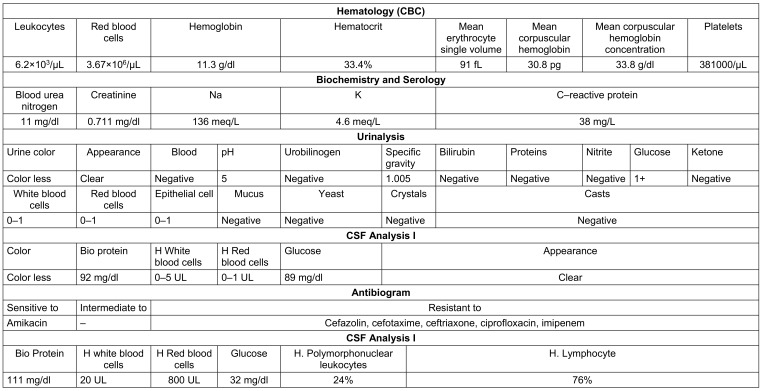
Laboratory test results
